# Addressing anti-black racism in an academic preterm birth initiative: perspectives from a mixed methods case study

**DOI:** 10.1186/s12889-023-16812-3

**Published:** 2023-10-18

**Authors:** Shira P. Rutman, Natasha Borgen, Solaire Spellen, Dante D. King, Martha J. Decker, Larry Rand, Alexis Cobbins, Claire D. Brindis

**Affiliations:** 1 Philip R. Lee Institute for Health Policy Studies, University of California, San Francisco, CA USA; 2 Department of Obstetrics, Gynecology and Reproductive Sciences, California Preterm Birth Initiative, University of California, San Francisco, San Francisco, CA USA; 3 Department of Epidemiology and Biostatistics, University of California, San Francisco, CA USA

**Keywords:** [MeSH] Racism, Evaluation, Organizational innovation, Premature birth; [Other terms] Anti-racism, Racial equity, Academic institutions

## Abstract

**Background:**

Growing recognition of racism perpetuated within academic institutions has given rise to anti-racism efforts in these settings. In June 2020, the university-based California Preterm Birth Initiative (PTBi) committed to an Anti-Racism Action Plan outlining an approach to address anti-Blackness. This case study assessed perspectives on PTBi’s anti-racism efforts to support continued growth toward racial equity within the initiative.

**Methods:**

This mixed methods case study included an online survey with multiple choice and open-ended survey items (*n* = 27) and key informant interviews (*n* = 8) of leadership, faculty, staff, and trainees working within the initiative. Survey and interview questions focused on perspectives about individual and organizational anti-racism competencies, perceived areas of initiative success, and opportunities for improvement. Qualitative interview and survey data were coded and organized into common themes within assessment domains.

**Results:**

Most survey respondents reported they felt competent in all the assessed anti-racism skills, including foundational knowledge and responding to workplace racism. They also felt confident in PTBi’s commitment to address anti-Blackness. Fewer respondents were clear on strategic plans, resources allocated, and how the anti-racism agenda was being implemented. Suggestions from both data sources included further operationalizing and communicating commitments, integrating an anti-racism lens across all activities, ensuring accountability including staffing and funding consistent with anti-racist approaches, persistence in hiring Black faculty, providing professional development and support for Black staff, and addressing unintentional interpersonal harms to Black individuals.

**Conclusions:**

This case study contributes key lessons which move beyond individual-level and theoretical approaches towards transparency and accountability in academic institutions aiming to address anti-Black racism. Even with PTBi’s strong commitment and efforts towards racial equity, these case study findings illustrate that actions must have sustained support by the broader institution and include leadership commitment, capacity-building via ongoing coaching and training, broad incorporation of anti-racism practices and procedures, continuous learning, and ongoing accountability for both short- and longer-term sustainable impact.

## Background

Academic institutions have begun to address racism and reckon with their policies and practices rooted in anti-Black ideology. This case study documents the approach, perspectives, and preliminary results of the California Preterm Birth Initiative (PTBi) at the University of California, San Francisco (UCSF) in confronting institutionalized anti-Blackness. Examining PTBi’s experiences contributes to the growing understanding of the ways academic institutions can meaningfully address anti-Black racism.

### Addressing anti-black racism within academic institutions

Racism, and more specifically, anti-Blackness, is one of the Unites States’ oldest and most insidious traditions [1]. Anti-Blackness is a sociopolitical system that devalues and dehumanizes people of African descent for the purpose of inequitable distribution of power and resources based on White supremacy [2, 3]. Academic institutions perpetuate anti-Black racism implicitly and explicitly through inequitable opportunities, funding, and representation; collective cultural community debasement; individual experiences of discrimination; and environments that fail to reflect and value Black cultures [4–6]. In June 2020, inspired by the increased public consciousness around anti-Blackness in the wake of the police murder of George Floyd, Ahmaud Arbery, Breonna Taylor, and others around the same time and many before them, two Black women in academia, Shardé M. Davis and Joy Melody Woods, coined the hashtag #BlackinTheIvory, amplifying experiences of anti-Black racism in higher education and highlighting the need for institutional reflection and change [7, 8]. The hashtag also responded to widespread concerns about universities sending out solidarity statements, which lacked action steps and methods for accountability.

The existing literature on institutional anti-racism approaches often focuses on the effectiveness of trainings aimed at individual-level change in attitudes and knowledge [9–12]. Yet, interventions focused solely on changing individual actions will not lead to the transformative progress needed [9, 13, 14]. Further, much of the literature presents frameworks and theories of change rather than operationalizing and assessing concrete anti-racism actions [10, 15]. Other areas of this literature focus on the prevalence of racism in direct-service settings, such as healthcare delivery and government organizations [9, 16]. Limited research has documented anti-racism approaches undertaken by academic initiatives or captured the perspectives of those most closely involved to assess directions and activities mid-course.

### California Preterm Birth Initiative

PTBi is a multi-pronged transdisciplinary research program that works in partnership with affected communities to reduce racial/ethnic disparities in preterm birth. Black birthing people experience preterm birth rates almost 50% higher than their White counterparts [18]. Since its establishment in 2016, PTBi has implemented several racial equity efforts, including providing racial equity trainings, increasing staff racial and ethnic diversity to reflect the communities served, incorporating anti-racism in communications, and prioritizing authentic community collaboration. During the national conversation around racism in June 2020, concurrent with the evolution of PTBi’s racial equity practices, the PTBi executive leadership team and communications staff developed and released an *Anti-Racism Action Plan* to publicly communicate their commitment to anti-racism approaches, which included nine goals for addressing anti-Blackness and advancing racial equity by the initiative [19]. To ensure accountability, the PTBi used this plan as a framework to assess their efforts.

The purpose of this case study is to document perspectives on the implementation of PTBi’s anti-racism approaches, which can inform further efforts to dismantle anti-Black racism through academic institutional change, as one of many sectors where changes are needed. Case studies contribute to our understanding of how theories and frameworks are experienced in practice. These applied and often real time works in progress, which fall outside the bounds of experimental examinations, acknowledge the importance of context and provide an operational, strategic alternative to actions solely guided by theory. Disseminating lessons learned from these studies can help promote the integration of more effective accountability mechanisms for sustained impact of addressing racism at academic institutions [17].

## Methods

### Program setting

PTBi is situated within UCSF School of Medicine’s Department of Obstetrics, Gynecology and Reproductive Sciences, with a perinatologist principal investigator and a strategic advisory board recruited to guide the initiative. Faculty directors oversee research portfolios within the individual PTBi focus areas of discovery and precision health; interventions across the reproductive life course; and collective impact and policy. Two staff directors also serve on the leadership team focusing on cross-cutting work to support the PTBi commitment to community engagement and research dissemination. The community engagement director supports a 25-member community advisory board and liaisons with community-based organizations; the director of communications supports communications and events. An external evaluation team documented the progress and challenges encountered in implementing key elements of the initiative.

### PTBi’s anti-racism efforts

Community partners identified issues of race and racism as a priority area as part of the evaluation in 2016–2017 [20]. In response, PTBi began a process to explicitly address racism as a root cause of racial/ethnic disparities in preterm birth rates through a mix of approaches that evolved over time [21]. Activities began with internal PTBi faculty and staff trainings, including a cultural humility workshop (2017) and an educational series on topics such as tokenism and the dynamics of power (2017-18). The initial series continued with monthly all-team racial equity trainings (2018), and then evolved into racial affinity group trainings focusing on discussion of Robin DiAngelo’s book “*White Fragility*” among people with White privilege (PWP) and an open discussion space for people of color (POC) (2019) [22]. In 2020, PTBi continued to integrate community partner recommendations to prioritize Black communities including implementing more comprehensive, in-depth racial equity trainings centered on White supremacy and anti-Blackness, and ways of confronting these in PTBi approaches and activities [3]. Changes to recruitment and hiring practices also shifted the composition of PTBi’s staff over time to a majority people of color, including the hiring of two Black women in the summer of 2020 (one of whom was promoted as a former community advisory board member) to lead the organization in partnership with the principal investigator as the executive leadership team.

### Study design

This case study used a mixed methods approach to capture perspectives on PTBi’s anti-racism efforts underway. In partnership with the PTBi executive leadership team, the external evaluation team translated PTBi’s nine *Anti-Racism Action Plan* goals into the following four assessment domains: (1) training and competencies, (2) organizational commitment, (3) practices, and (4) external partnerships and communications, which are outlined in Table 1. Examples of PTBi’s anti-racism efforts are provided for each domain.

**Table 1:** PTBi anti-racism goals, examples of anti-racism activities, and case study domains

PTBi anti-racism goals	Example anti-racism activities	Domain
Continue to be constantly aware of the trauma our Black colleagues and community members face on a daily basis	• *Hosted in-depth bi-monthly racial equity trainings* • *Facilitated leadership racial equity assessments*	Trainings and competencies
Ensuring our whole staff recognize the great power that each one of us hold in inspiring and creating change for good	• *Provided support through racial affinity group training sessions*	
Developing more ways to support and hold space for our Black colleagues, community advisory board members and partners	• *Created protocol for community research summaries and inclusion of community members as co-authors on academic publications*	Organizational commitment
Continue elevating strength, resilience, and an asset-based framework	• *Launched community-designed public awareness campaign with strengths-based focus on Black and POC birth* • *Funded evaluation of anti-racism activities*	
Tightening our focus on Black-led and Black-focused birth research	• *Recruited more Black postdoctoral fellows* • *Focused funding on Black community*	
Continue hiring more Black staff and faculty	• *Recruited more Black postdoctoral fellows also supporting a faculty pipeline* • *Hired Black Associate Director*	Practices
Continue elevating and championing the voices and work of Black team members, researchers, providers, and advocates at UCSF and at-large	• *Transitioned more community-based staff from contract to career positions* • *Allocated professional development funds for all staff*	
Continue calling out racist systems and educating non-Black researchers and academics on how they can begin to dismantle racism within their research programs and organizations	• *Created a value statement about commitment to racial equity* • *Shared lessons learned about anti-racism practices with partners* • *Supported community-led research symposia and events*	External partnerships and communications
Continue developing our community workforce and capacity building programs and hiring from the communities we invest in whenever we can	• *Contracted with primarily POC-led businesses* • *Hired former community-partner as Executive Director*	

PTBi, California Preterm Birth Initiative; POC, People of color; UCSF, University of California, San Francisco

The issue of potential bias when authors examine their own institution is a valid concern in research. PTBi and external evaluators are within the same university, but are housed under separate research institutes. To establish independence in the study and ensure a critical and impartial perspective, PTBi faculty and staff (including affiliated authors) were not involved in the collection or analysis of data, and evaluators only shared selected de-identified data with PTBi.

### Data collection

The case study included a survey and key informant interviews with PTBi leadership, faculty, staff, and trainees. Close-ended survey items included participants’ self-assessment of their individual and PTBi’s organizational anti-racism competencies, perceived areas of initiative success, and opportunities for improvement. Participants were invited to share their perspectives about the influence of PTBi’s racial equity trainings, PTBi’s work environment, and overall recommendations through their responses to open-ended questions. The survey also asked participants questions about their racial/ethnic identity and their length of involvement and role within the PTBi. Online surveys were sent to all PTBi leaders, faculty, staff, and trainees (*N* = 36) in December 2020 using Qualtrics, a secure online data collection platform [23].

To support data source triangulation, evaluators used purposive sampling to invite key informants representing different organizational roles, length of PTBi involvement, and racial/ethnic identities (based on PTBi racial equity training affinity groups of POC and PWP) to participate in confidential, semi-structured interviews [24]. Open-ended interview questions were designed to further elucidate and support interpretation of survey topics. Evaluators conducted an initial set of four interviews, and after consideration of information captured, conducted another four interviews, during which consistent themes and examples were repeated across interviews. Reaching theme saturation points, it was determined that insights from key informants were adequately included (*n* = 8) [25]. Interview and survey introductions provided assurances of confidentiality and explained the voluntary nature of participation. Interviews were conducted between January and April of 2021 and averaged 60-minutes in length. Interviews were audio-recorded and transcribed. The UCSF Institutional Review Board determined that as a quality improvement activity, the evaluation did not require institutional human subjects’ review and approval.

### Data analyses

Survey data were downloaded into Microsoft Excel for analysis [26]. Evaluators conducted descriptive analyses of response frequencies across all respondents and examined differences by affinity group (POC or PWP). The sample was too small to perform statistical tests of significance between these groups.

For interview data, evaluators utilized both structural and emerging coding for qualitative analysis, creating a list of codes based on the domains, and refining the list during the coding process [27]. Two transcripts were double coded for inter-coder consistency with an inter-rater reliability kappa level of 0.80 using a Pooled Cohen’s Kappa coefficient and Cohen’s Kappa for each of the codes included in the test. During the coding process, the evaluators discussed code application, definitions, and coding commonalities and discrepancies. Evaluators examined how often themes occurred in the data and compared across different racial/ethnic identities. They also reviewed for consistency in themes across interview and survey data. Transcriptions were coded and analyzed using Dedoose online software version 8 [28].

## Results

We received 27 survey responses (response rate 75%). The diversity of survey respondents reflected the overall racial and ethnic composition of the PTBi. Over one-third of survey respondents identified their race/ethnicity as Black or African American (37%; *n* = 10), one-third as White (33%; *n* = 9), and almost one-third as a race/ethnicity other than White, including Asian, Native Hawaiian/Pacific Islander, Hispanic/Latinx, and two or more races/ethnicities (30%; *n* = 8). The sample was also representative of the demographic make-up within PTBi organizational roles. 60% of survey respondents who represented initiative leaders, faculty, or supervisors (37%, *n* = 10) identified as Black or African American (*n* = 6); and 71% of survey respondents representing staff, post-doctoral trainees, or students (63%, *n* = 17) identified as a race and/or ethnicity other than White (*n* = 12). This section summarizes results from all data sources within each assessment domain.

### Training and competencies

The first domain included respondents’ perspectives about PTBi’s racial equity trainings and a self-assessment of their anti-racism competencies.

#### Trainings

Though most survey respondents indicated that they were either somewhat or very familiar with topics related to racial equity and anti-Blackness prior to participating in the PTBi trainings (*n =* 25; 93%), a majority found the monthly trainings to be useful (*n* = 24; 89%). Almost all who were very familiar with the topics were POC (*n* = 12; 44%). The most common responses about the impact of the trainings related to evolution in interactions with colleagues, increased self-reflection, sense of empowerment, and institutional accountability to voice and respond to concerns from POC.

I have been able to better recognize the impact of our White supremacist culture on several of my Black co-workers, which has allowed me to not only examine my role in perpetuating it, but also offer my Black co-workers greater support and understanding. [PWP]

Other survey comments on the impact of the trainings included incorporating the use of anti-racist language and approaches within research activities, and changes in recruitment and hiring practices. A few respondents shared concerns about the impact of the trainings on POC and the need for emotional support, an issue which was also reflected in a few interviews (Table 2). One interviewee (PWP) recommended that PTBi support building personal connections to address team tensions, which emerged because of the issues that were raised during the trainings.

**Table 2:** PTBi anti-racism case study domains, topics, and illustrative quotes

Domain	Topic	Illustrative quotes
Trainings and competencies	Trainings	*It could be re-traumatizing sometimes going through those things, and I don’t know that necessarily the White colleagues understand that to the same kind of depth or level.* [POC]
	Competencies	*[White colleagues are] all for [anti-racism] in the moment of the racial equity training, but it maybe doesn’t come to fruition in practice when dealing with staff or community or other colleagues.* [POC]
Organizational commitment	Leadership	*We have to be willing to turn the mirror on ourselves and say, “Well, where is it that we are participating in White supremacist structures?”* [POC]
	Strategy and committed resources	*Things just aren’t transparent all the time. They’re getting better, but in terms of the budget, the staff was kind of not aware of how much different teams are spending… I think, sometimes there’s an assumption that the staff knows what’s going on.* [POC]
	Inclusive environment	*I am figuring out where I fit in terms of anti-Blackness as someone who is racially ambiguous, and how people treat me because of that. And figuring out when I should speak up on behalf of some of the Black people or help them elevate their voices or am I going to take a step back.* [POC]
Practices	Recruitment and hiring	*The staff was really disappointed [about PTBi’s search for Black faculty]. Because it was like, “We’re looking for someone,” and then we never hired anyone, and the staff never really got an update about that at all… So, people were like, “Are we still committed to [hiring Black faculty]?”* [POC]
	Professional development	*It would be nice to have time to really think about, “What do I want to do next and how can PTBi help with that?” Whether that’s doing some trainings or whatever… Figuring out [university] pathways or in other places.* [POC]
	Integrating racial equity strategies	*[PTBi] does a great job at calling things out such as racism and needs more work to operationalize how the initiative will be focusing on aligning current research projects and goals, and allocation of resources to align with its new mission.* [POC]
External partnerships and communications	External communications	*[We need to] articulate why we are focusing on anti-Blackness and not other forms of anti-racism. I agree with this approach, but I do not believe that it has been strongly articulated.* [POC]
	Partnerships	*We looked at what percentage of our funds go towards community partnership [efforts], and it was 5%, which really doesn’t sound very good.* [PWP]

PTBi, California Preterm Birth Initiative; POC, Person of color; PWP, Person with White privilege

#### Competencies

Most survey respondents agreed they felt competent in five of the six assessed competencies (Range 81-96%) (Table 3). However, only about half felt capable of discussing anti-Blackness and racism with colleagues (*n* = 14; 52%), and a few (PWP and POC) described the ability to address microaggressions as an area for needed improvement in open-ended survey questions. This was also reflected in several interviews (Table 2). Interviewees from both racial affinity groups also shared the need for PWP to be more open to receiving feedback, and that empathy and emotional investment were necessary for change.

**Table 3:** PTBi anti-racism survey indicators in descending agreement: Competencies and Organizational commitment domains (*N* = 27)

	Agree	Disagree	Not sure
**Competencies**	*n* (%)	*n* (%)	*n* (%)
I understand America’s legacy of racial inequity and anti-Blackness and its link to health outcomes.	26 (96)	0 (0)	1 (4)
I know how to recognize anti-Blackness and racism in my workplace.	25 (93)	1 (4)	1 (4)
I believe that PTBi faculty and staff have a basic understanding of racial equity, including anti-Black racism.	25 (93)	1 (4)	1 (4)
I know what to do when I see anti-Blackness and racism playing out at work.	25 (93)	1 (4)	1 (4)
It is my responsibility to advance racial equity and address anti-Blackness in my work.	22 (81)	4 (15)	1 (4)
I feel capable of discussing anti-Blackness and racism at work with my colleagues.	14 (52)	5 (19)	8 (30)
**Organizational commitment**	*n* (%)	*n* (%)	*n* (%)
PTBi leadership has demonstrated a commitment to advancing racial equity and addressing anti-Blackness.	25 (93)	2 (7)	0 (0)
PTBi leadership is equipped to participate in internal and external conversations around racial equity and anti-Blackness.	21 (78)	4 (15)	2 (7)
PTBi fosters an environment in which the full identities of individuals, different cultures, racial, ethnic, and educational backgrounds, as well as different sexual orientations, ages, and genders are valued and respected.	21 (78)	4 (15)	2 (7)
PTBi prioritizes funding to support Black-led community-based research.^**a**^	12 (46)	8 (31)	6 (23)
PTBi has monitoring and evaluation plans, including indicators of progress, to measure its impact on advancing racial equity and addressing anti-Blackness internally.^**a**^	10 (38)	9 (35)	7 (27)
PTBi has strategies in place to advance racial equity and address anti-Blackness with clear actions, roles, and responsibilities.^**a**^	9 (35)	11 (42)	6 (23)
PTBi has committed sufficient allocation of resources, including staff time, to advancing racial equity and addressing anti-Blackness.^**a**^	8 (31)	12 (46)	6 (23)

PTBi, California Preterm Birth Initiative

^**a**^*N* = 26; one survey response missing

The White management —and also colleagues, employees—they have to be more accountable about owning it, and not being defensive, but listening, and humbling themselves. [POC]

### Organizational commitment

The second domain assessed leadership commitment and capacity to engage in issues of racial equity and anti-Blackness, strategic plans and committed resources, and fostering an environment that values and respects individuals’ full identities.

#### Leadership

Most survey respondents agreed that PTBi leaders demonstrated a commitment to advancing racial equity and addressing anti-Blackness (*n* = 25; 93%). However, fewer agreed that leaders were equipped to participate in internal and external conversations on these issues (*n* = 21; 78%) (Table 3). A few interviewees described the need for PTBi to address racial power dynamics, particularly anti-Blackness, as reflected in the academic hierarchy overall and the PTBi’s current leadership and staffing (Table 2).

We are a hierarchical organization within the institution, so that’s where a lot of the push comes from… because we’re still a group of White directors, of White faculty, and all the other directors who are of color are not faculty. [PWP]

#### Strategy and committed resources

Only about a third of survey respondents agreed (*n* = 9; 35%) that PTBi had strategies in place and committed sufficient resources to advancing racial equity and addressing anti-Blackness (Table 3). One interviewee explained:

[We need to] make sure that a significant portion of the budget actually goes to facilitate the equity ideas. I know a lot of our staff and faculty have talked about how the budget may not reflect our commitment yet, and that more of it should be allocated to serving our communities and less so to maybe more academic initiatives. [PWP]

A couple of interviewees also noted both a lack of transparency in decision-making and resource allocation, as well as recognizing improvements that had taken place in this area (Table 2). Less than half of survey respondents agreed PTBi prioritizes funding Black-led community-based research (*n* = 12; 46%), while 23% (*n* = 6) reported they were not sure (Table 3). However, one interviewee described a shift in PTBi’s post-doctoral fellowship towards primarily Black scholars. A couple of survey respondents also noted that more buy-in and sustainable commitment to anti-Black racism efforts across the university would further support the PTBi’s programmatic racial equity goals.

#### Inclusive environment

Over three-quarters of survey respondents agreed (*n =* 21; 78%) that PTBi fosters an inclusive environment that values and respects individuals’ full identities (Table 3); however, a much smaller percentage of POC compared to PWP respondents agreed (*n* = 6; 35% and *n* = 6; 67% respectively, data not in tables). A few interviewees expressed their desire to see PTBi address parallel and interrelated systems of oppression, such as capitalism, sexism, transphobia, and barriers based on educational background. In addition, one participant expressed that, “More attention [should be] paid to the views and struggles of non-Black POCs,” and another POC felt challenged in finding their place within the anti-Black racism efforts (Table 2).

### Practices

The third domain included perceived changes in PTBi’s anti-racist practices, such as prioritizing recruitment and hiring of Black individuals, providing professional development, and integrating anti-racism efforts into job performance objectives and evaluations of staff and faculty.

#### Recruitment and hiring practices

59% (*n* = 16) of survey respondents agreed that PTBi prioritized recruitment and hiring of Black individuals and several interviewees commented on this with one POC noting, “Our demographics have changed drastically.” However, 15% (*n* = 4) disagreed and 26% (*n* = 7) were unsure (Table 4). A couple of survey respondents and a few interviewees specifically noted the need to hire Black faculty (Table 2). One interviewee explained the challenge of recruiting Black junior faculty and post-doctoral trainees into positions within the PTBi, due to it being an initiative with almost all White faculty.

**Table 4:** PTBi anti-racism survey indicators in descending agreement: Practices and External partnerships and communications domains (*N* = 27)

	Agree	Disagree	Not sure
**Practices**	*n* (%)	*n* (%)	*n* (%)
I understand my role in advancing racial equity and addressing anti-Blackness within the PTBi.	22 (81)	4 (15)	1 (4)
I am familiar with the ways the PTBi is implementing its commitments to advancing racial equity and addressing anti-Blackness.^**a**^	15 (60)	6 (24)	4 (15)
There are opportunities within the PTBi for individuals from underrepresented racial groups to hold positions of leadership and decision-making.	23 (85)	3 (11)	1 (4)
PTBi prioritizes the recruitment and hiring of Black-identified individuals, including for all funded studies and projects.	16 (59)	4 (15)	7 (26)
There are opportunities within the PTBi for individuals from underrepresented racial groups to receive mentorship, coaching, and support.	13 (48)	6 (22)	8 (30)
PTBi incorporates racial equity knowledge, skills, and practices into performance objectives (such as job descriptions and work plans), and evaluations for staff and faculty.	7 (26)	14 (52)	6 (22)
**External partnerships and communications**	*n* (%)	*n* (%)	*n* (%)
I believe that PTBi’s funded partners have a basic understanding of racial equity, including anti-Black racism.	25 (93)	0 (0)	2 (7)
I can articulate PTBi’s commitment to advancing racial equity and addressing anti-Blackness to external partners.^**a**^	21 (84)	2 (8)	2 (8)
PTBi makes deliberate efforts to build the leadership capacity of community-based partners to advocate for issues related to racial equity and anti-Blackness.	14 (52)	5 (19)	8 (30)
PTBi dedicates sufficient resources to external communications focused on advancing racial equity and addressing anti-Blackness.^**b**^	12 (46)	8 (31)	6 (23)

PTBi, California Preterm Birth Initiative

^**a**^*N* = 25; two survey responses missing

^**b**^*N* = 26; one survey response missing

#### Professional development

While most survey respondents agreed that there were opportunities for individuals from underrepresented racial groups to hold positions of leadership and decision-making within PTBi (*n* = 23; 85%), less than half felt there were adequate opportunities for mentorship, coaching, and support (*n* = 13; 48%). Nearly a third, (*n* = 8; 30%) were not sure if the existing opportunities were adequate (Table 4). Several respondents commented on the need for improvement in professional development, including supporting university career advancement pathways for staff.

I just don’t think in terms of retention, [that the leadership/faculty] think about what needs to change about our environment to make people of color want to stay here. [POC]

#### Integrating racial equity strategies

Overall, more than half of survey respondents disagreed (*n* = 14; 52%) and 22% (*n* = 6) were not sure whether PTBi successfully incorporates racial equity knowledge, skills, and practices into job performance objectives and performance evaluations of staff and faculty (Table 4). However, this assessment differs by racial affinity group. Most POC respondents either disagreed or were unsure whether this integration of racial equity strategies was actually taking place within PTBi (*n* = 15; 94%) compared to one-third of PWP respondents (*n* = 6; 33%) (data not in tables).

Internal communications about PTBi’s implementation of its anti-racism goals also surfaced in survey responses with a large majority reporting they understood their own role in advancing racial equity and addressing anti-Blackness within PTBi (*n* = 22; 81%). In rating PTBi’s efforts, more than half reported that they were familiar with the ways the initiative implements these commitments (*n* = 15; 60%). A few survey respondents recommended PTBi further operationalize their goals, including specifying alignment of strategies, projects, and resources (Table 2).

A survey question asked respondents to check all that apply from a list of supportive factors that they would need to individually pursue anti-racism strategies more actively. The most frequent responses included more funding to promote solutions, assuring sustainability of racial equity efforts, and dedicated staff time provided to racial equity efforts. The need for more information and for more training were the least common responses (data not in tables). Staff and leadership interviewees also noted the challenges of integrating anti-racism strategies into their day-to-day work, such as in communications or research methods (Table 2).

I do feel like everything has been focused on our racial equity trainings… and not as much like our day-to-day tasks. [POC]

A few interviewees described a differential burden on POC for integrating anti-racism into PTBi’s efforts explained as disparate levels of commitment, a lack of dedicated time in which POC accomplished such tasks, and a lack of expertise among White colleagues.

As great as [the White leaders and staff] are, their training is not in racial equity. [POC]

### External partnerships and communications

Less than half of survey respondents agreed (*n* = 12; 46%) that PTBi dedicates sufficient resources to external communications focused on advancing racial equity and addressing anti-Blackness, while 23% (*n* = 6) were not sure (Table 4). A couple of interviewees also described the need to better articulate PTBi’s anti-racism strategies in its community partnership building and communication efforts (Table 2).

Several interviewees acknowledged the importance of community partnerships in PTBi’s efforts to address anti-Black racism. However, only 52% (*n* = 14) of survey respondents agreed and 30% (*n* = 8) were unsure whether PTBi makes deliberate efforts to promote community-based partner leadership in advocating for racial equity and addressing anti-Blackness (Table 4). One staff person commented on the evolution of activities to incorporate community partners more, including writing research summaries for and reporting back research findings to community partners. Two interviewees commented on insufficient staffing for community partnership activities (Table 2).

It makes no sense to me that [PTBi have only] two full-time staff members for [community engagement efforts] in three geographies. [POC]

## Discussion

This case study provides a rare assessment of an academic research initiative’s efforts to directly confront the issue of anti-Black racism not only in its goals for addressing racial/ethnic disparities in preterm birth outcomes, but in its approach to those efforts at the individual- and initiative-wide levels. Studies of anti-racism efforts seldom include participant assessment of initiative-level approaches. These results highlight the need for greater transparency, communication, and accountability supported through dedicated funding and infrastructure to focus on these assessments [29]. PTBi’s relatively large and flexible philanthropic funding source created an opportunity for the racial equity journey the initiative pursued, which diversified and expanded in strategy over time as additional needs were identified [30, 31]. PTBi’s commitment to sustained, meaningful change was further strengthened through reflecting on these efforts. Black scholars have shared recommended anti-racist actions specifically for the academic community, which we reference throughout the following discussion as relevant to each of our case study domains [4, 32, 33].

Participants overall had positive opinions of PTBi’s racial equity trainings, including improvements in institutional accountability to voice and respond to concerns from POC. They also recommended strategies for healing and avoiding triggering racial trauma for POC because of the training process. Even with a high-level of self-assessed anti-racism competencies and overall listing additional training as lower priority than other areas to advance individual anti-racism strategies, participants described the need for additional support to engage in discussions about anti-Blackness and addressing workplace racial microaggressions. Dialogue about racial microaggressions can be particularly challenging in academic institutions, as most of these institutions are led by and oriented to PWP [34]. Authors of a pivotal microaggressions framework recommend addressing the distinct fears of well-intentioned PWP of appearing racist, recognizing one’s own racism, acknowledging White privilege, and accepting the consequences of one’s behavior [34]. Research on workplace psychological safety recommends creating an environment where POC do not fear being humiliated, ignored, or blamed [35]. Suggested strategies to promote this type of environment include supporting perspective-taking and empathy-building for a growth mindset of lifelong learning [35]. Implementing ongoing implicit bias and diversity training for all individuals at the institution will support these aims [33]. Within PTBi, providing additional “safe” venues for each racial affinity group to share and express their concerns also helped the dialogues that occurred within and between racial groups.

The theme of racial power dynamics within academic institutions surfaced, as almost all PTBi faculty positions were held by PWP despite ongoing feedback about the need for PTBi to hire Black faculty, and ineffective attempts to do so. While PTBi’s university home has taken steps to address anti-Blackness, PTBi was on the forefront of this work within the university, and study participants noted that increased and sustainable commitment across the university would also help create the type of environment needed to achieve PTBi’s racial equity goals. University-wide initiatives for faculty of color recruitment and retention are necessary to support institutional culture changes, signal to departments and initiatives that anti-racism changes are viewed by the university as imperative, and to incorporate a layer of additional departmental accountability [10]. However, improving strategies for Black faculty recruitment and retention will require moving beyond symbolic gestures with multi-pronged, innovative approaches in concert with university-wide commitments [36]. For recruitment and retention of Black faculty, these should include commitment to sustained, non-traditional recruitment efforts; identification of specific potential environmental barriers (e.g., cost of living, housing, local schools, etc.); examination of socio-cultural concerns of Black faculty, establishment of strategic partnerships with pathway institutions and post-doctoral training opportunities for Black faculty candidates, and incorporation of anti-Black racism efforts into promotion, tenure, and annual review processes [33].

Another key finding was the importance of operationalizing and committing resources to anti-racism goals and ongoing internal communications across all roles on the strategies undertaken. Specific areas recommended for resource allocation included professional development for Black and other POC, expanded external communications, including campus-wide, on anti-Black racism efforts, and increased staffing for community partnership activities.

In the assessment of PTBi’s practices in relation to its anti-racism goals, most participants acknowledged efforts to increase staff racial representation, which is essential for any academic institution and others pursing racial equity. Several participants also pointed to areas for improvement, including fostering an environment that values staff of color and providing support for staff to pursue opportunities for advancing their university career pathways. Creating a climate where staff of color feel valued for their unique contributions, supported in their career development, and connected to their co-workers can lead to a sense of belonging and improved work experiences [32]. Comments about the need for the initiative to expand the inclusion of people from historically underrepresented and intersecting identities revealed an opportunity for further programmatic training regarding how anti-Blackness interacts with and is embedded in other systems of oppression, including gender and sexual identity [3].

Participants recommended PTBi further incorporate racial equity into every facet of the work, which would also enhance team chemistry, intentionality, and sustainability. Participants felt supervisors should ensure that anti-racism activities are equitably distributed across the organization to remove the undue burden on POC to always initiate and facilitate anti-racism actions. This undue burden has been explained by some as a “tax,” including a disparity in responsibility for achieving diversity efforts, as well as in experiences of racism, isolation, and inequities in receipt of mentorship, clinical versus scholarly opportunities, and promotions [37]. PTBi and other academic institutions, can draw on best practices outlined by its university home for performance reviews that integrate identified anti-racist objectives and metrics that are measurable and can produce tangible outcomes for all levels of staff, trainees, and faculty [36]. Academic leadership can further promote wellness and self-care among POC team members to help offset the impact of generations of systemic racism, by providing self-care information, training, and resources, and implementing programs that respond to the specific wellness needs of Black individuals [38, 39].

### Limitations

This case study has some limitations. Our data collection tools assessed PTBi’s combined efforts to “address anti-Blackness and advance racial equity,” therefore, our ability to distinguish the topic of anti-Blackness from other anti-racism efforts within the initiative may be limited. Due to our case study sample size, to protect participant confidentiality, we were limited in our ability to present disaggregated results within specific racial/ethnic groups, professional positions, and length of involvement in PTBi. Where possible, future studies may benefit from analysis of respondent characteristics or other variables to understand the dynamics being examined. This study would also be strengthened by the collection of follow-up data to examine changes over time as the program incorporated additional activities as part of its quality improvement efforts. Despite these limitations, this case study provides valuable insights regarding the strategies and challenges of addressing anti-Black racism within an academic setting, as well as the value of investing in efforts to document the barriers, as well as facilitators, that help advance such an agenda.

## Conclusions

Even with PTBi’s strong commitment, actions, and progress towards racial equity, this case study illustrates that academic institutions’ efforts to systematically address racism at the individual and institutional-levels must be supported by leadership commitment, capacity-building, anti-racism practices and procedures, continuous learning, and accountability within individual academic initiatives, as well as throughout the institution. To advance racial equity and address anti-Blackness, it is critical for academic institutions to continuously examine the racist systems through which they were developed and continue to operate, and to dedicate themselves to pursuing transformative actions.

## Data Availability

The datasets generated and analyzed during the current study are not publicly available due to confidentiality concerns and the complexity of creating de-identified data. For data related queries kindly contact shira.rutman@ucsf.edu. The data collection tools, interview guide and survey, are available upon email request to the corresponding author.

## Abbreviations

POC: People of color
PWP: People with White privilege
PTBi: California Preterm Birth Initiative
UCSF: University of California, San Francisco
